# Optimal Blend Between Fluorinated Esters and Fluorinated Ether for High-Performance Lithium-Ion Cells at High Voltage

**DOI:** 10.3390/ma18020274

**Published:** 2025-01-09

**Authors:** Yong Sheng, Bo Liu, Junjiang He, Maoyong Zhi, Dongxu Ouyang

**Affiliations:** 1 China Academy of Safety Science and Technology, Beijing 100012, China; 18910181582@163.com (Y.S.); hjj0513@mail.ustc.edu.cn (J.H.); 2 College of Safety Science and Engineering, Nanjing Tech University, Nanjing 211816, China; 202261201047@njtech.edu.cn; 3 Sichuan Province All-Electric Navigation Aircraft Key Technology Engineering Research Center, Civil Aviation Flight University of China, Guanghan 618307, China; zhimaoyong@cafuc.edu.cn

**Keywords:** lithium-ion cell, fluorinated ester, fluorinated ether, high voltage, safety

## Abstract

An experimental investigation is conducted to identify the optimal blend of fluoroethylene carbonate (FEC), 3,3,3-trifluoropropylene carbonate (TFEC), and various fluorinated ethers, including 1,1,2,2-tetrafluoroethyl-2,2,2-trifluoroethyl ether (HFE), 1,1,2,2-tetrafluoroethyl-2,2,3,3-tetrafluoropropyl ether (TTE), and bis(2,2,2-trifluoroethyl) ether (BTE), to enhance the performances of lithium-ion cells at high voltage. The cell incorporating TTE exhibits a significantly superior capacity for retention after long-term cycling at 4.5 V, which might be attributed to the improved kinetics of lithium ions and the generation of a thin, reliable, and inorganic-rich electrode–electrolyte interface. This enhancement facilitates greater lithium ion mobility within the cell, while effectively suppressing active lithium loss and side reactions between the electrodes and electrolytes at elevated voltages. Furthermore, the cell with TTE demonstrates a superior rate capability and high-temperature performance. As a result of the inherent safety characteristics of these all-fluorinated electrolytes, cells using these formulations show excellent safety properties under typical abuse scenarios. Except at elevated temperatures, none of the cells undergo thermal runaway when subjected to mechanical or electrical abuse, and there are minimal differences in safety performance across the different formulations. Considering electrochemical performance, safety, and cost factors, it can be concluded that TTE might be more optimal to cooperate with FEC and TFEC for high-performance high-voltage cells.

## 1. Introduction

Fluorinated and even all-fluorinated electrolytes have garnered significant attention for enhancing lithium-ion cell performance at high voltages [1,2,3]. Benefiting from the strong electron-absorbing capacity and high electronegativity of the fluorine element, these electrolytes typically illustrate improved antioxidant features, enabling them to operate effectively at high voltages. Additionally, the presence of fluorine in the molecular structure helps suppress the electrolyte’s combustibility, further promoting the safety of the cells [4].

Fluorinated solvents or additives are increasingly used to replace traditional carbonate-based electrolytes, resulting in a diverse range of fluorinated electrolyte formulations. Among these, fluorinated esters are particularly popular and can be categorized into cyclic and linear compounds based on their molecular structures [5,6,7,8,9,10,11,12,13,14,15,16,17,18]. Representative cyclic fluorinated esters include fluoroethylene carbonate (FEC), difluoroethylene carbonate (DFEC), and 3,3,3-trifluoropropylene carbonate (TFEC), where the hydrogen atoms of ethylene carbonate (EC) are replaced by one fluorine atom, two fluorine atoms, and one trifluoromethyl group, respectively. Common linear fluorinated esters, such as methyl(2,2,2-trifluoroethyl) carbonate (FEMC), ethyl(2,2,2-trifluoroethyl) carbonate (FEEC), and bis(2,2,2-trifluoroethyl) carbonate (FEBC), are derived from the fluorination of ethyl methyl carbonate (EMC) and diethyl carbonate (DEC). For instance, Zhang et al. [5] proposed an all-fluorinated electrolyte by replacing EC and dimethyl carbonate (DMC) with FEC and FEEC, respectively, to improve cell performance at high voltages. This electrolyte optimized lithium ion solvation environments and generated stable, LiF-rich interfaces, leading to a capacity retention of 72.3% post 225 cycles at 4.6 V and an average Coulombic efficiency of 99.8% for LiNi_0.8_Mn_0.1_Co_0.1_O_2_ (NMC811) cells. Given that an increasing substitution of fluorine within cyclic carbonates might facilitate the production of a protective passivation layer on the anode, DFEC was introduced by Su et al. [6] as a co-solvent with EMC for high-voltage LiNi_0.6_Mn_0.2_Co_0.2_O_2_ cells. This electrolyte effectively mitigated cell degradation, achieving a capacity retention of over 82% and an average Coulombic efficiency of 99.95% post 400 cycles charged to 4.4 V. In contrast, a blend of FEC and EMC yielded only 31% capacity retention and a Coulombic efficiency of 99.73%. Selecting FEC, EMC, and FEBC, Zhang et al. [7] developed a inflammable and low-exothermic electrolyte, which efficiently reduced the peak temperature and the peak temperature rising rate of lithium-ion pouch cells in Accelerating Rate Calorimetry (ARC) tests. In addition, a competitive capacity retention of 80% after 200 cycles for NMC811 cells was also demonstrated when charged to 4.4 V, benefitting from the sound compatibility between the newly proposed electrolyte and lithium metal.

On the other hand, fluorinated ethers are widely utilized in electrolytes to expand the electrochemical window and enhance the solvation structure, ultimately improving the high-voltage property of lithium-ion cells [19,20,21,22,23,24,25,26,27,28,29,30,31]. Common fluorinated ethers involve 1,1,2,2-tetrafluoroethyl-2,2,3,3-tetrafluoropropyl ether (TTE), bis(2,2,2-trifluoroethyl) ether (BTE), 1H,1H,5H-perfluoropentyl-1,1,2,2-tetrafluoroethylether (PTE), and 1,1,2,2-tetrafluoroethyl-2,2,2-trifluoroethyl ether (HFE). Wu et al. [14] reported a sulfone–HFE blended electrolyte for high-voltage NMC811 cells. This electrolyte, which featured an anion-dominated solvation structure and a robust, inorganic-rich electrode–electrolyte interface, resulted in a remarkable capacity retention of 88% post 1000 cycles with an upper cutoff voltage of 4.5 V. Additionally, the thermal safety of the cells was superior to that of cells with conventional electrolytes, with the self-heating and thermal runaway temperatures increased by 9.6 °C and 42.0 °C, respectively. Hou et al. [15] incorporated 1.0 M lithium bistrifluoromethosulfonimide (LiTFSI) into a solvent blend of sulfone, TTE, and FEC in a 0.475:0.475:0.05 volume ratio. This electrolyte exhibited an impressive electrochemical window of 5.1 V, contributing to a capacity retention of 85% after completing 150 cycles (0.5C rate) in the 3.0–4.7 V range for NMC811 cells. The authors attributed these improvements to enhanced structural and interfacial stability at the cathode, as well as reduced dendrite growth and improved lithium plating/stripping on the anode. In another study, Hai et al. [16] highlighted the critical role of fluorinated ethers in enhancing the high-voltage performance of LiNi_0.5_Mn_1.5_O_4_ cells. They investigated five fluorinated ethers, i.e., ethyl 1,1,2,2-tetrafluoroethyl ether (ETE), HFE, TTE, 1,1,2,3,3,3-hexafluoropropyl-2,2,2-trifluoroethyl ether (HTE), and PTE, to blend with sulfone and lithium hexafluorophosphate (LiPF_6_). The study found that long-chain and fluorine-rich fluorinated ethers like HTE and PTE promoted the dissociation of electrolyte clusters and favored the formation of inorganic-rich passivation layers on the anode, leading to superior electrochemical performance. Cells using HTE and PTE showed capacity retentions of 89.4% and 83.2%, respectively, after 500 cycles at 0.5 C, charged to 5.0 V.

In previous work, the authors developed an all-fluorinated electrolyte composed of fluorinated esters (FEC and TFEC) and a fluorinated ether (TTE) [32], which significantly promoted the electrochemical and safety feature of cells charged to 4.5 V. This raises an important question: which other fluorinated ethers, besides TTE, synergize well with the FEC-TFEC blend, and what is the optimal formulation of FEC, TFEC, and fluorinated ether for high-performance, high-voltage lithium-ion cells? With the rapid development of lithium-ion cell technologies, there are more and more novel fluorinated ethers proposed; however, the optimal blend between fluorinated esters and fluorinated ether for high-performance lithium-ion cells at high voltage is still waiting to be unveiled. In the present study, three representative fluorinated ethers (TTE, HFE, and BTE) were selected as co-solvents for FEC and TFEC, resulting in three all-fluorinated electrolytes. The electrochemical and safety performance of cells utilizing these specific all-fluorinated electrolytes were systematically evaluated and compared to identify the best combination of FEC, TFEC, and fluorinated ether. Additionally, the operational mechanisms of the electrolytes were explored through computational simulations and material characterization.

## 2. Experimental Procedure

### 2.1. Electrolyte Preparation

Three types of all-fluorinated electrolytes were prepared using FEC, TFEC, and one fluorinated ether (TTE, HFE, or BTE) as solvents in a 2:6:2 weight ratio, with 1.5 M LiPF_6_ as the lithium salt. These formulations were designated as FTT, FTH, and FTB, respectively. The solvent ratio was determined by referring to Fan’s work [33], where a blend between fluorinated ester and fluorinated ether was proposed. All these solvents and the salt were provided by Hunan Avenue New Material Co., Ltd. (Xiangtan, China), with a purity greater than 99%.

### 2.2. Coin Cell Preparation

Coin cells were assembled in a glove box (MIKROUNA SUPER) filled with nitrogen. Each cell consisted of an NMC811 cathode (composed of NMC811/carbon/binder with a ratio of 92:4:4 by weight and a diameter of 12 mm), a lithium metal anode (with a diameter of 12 mm), a Celgard separator (2325, composed of PP/PE/PP with a thickness of 25 μm, porosity of 40%, and transverse direction shrinkage rate of 0%), and one of the prepared electrolytes (FTT, FTH, or FTB). All of these were provided by Guangdong Canrd New Energy Technology Co., Ltd., (Dongguan, China). The NMC811 cathode had a loading of 8.3 mg/cm^2^, with a specific capacity of ca. 188 mAh/g charged to 4.3 V. About 60 μL of electrolyte was added to each cell. The cells were then subjected to electrochemical testing, as outlined in Section 2.4.

### 2.3. Pouch Cell Preparation

Dry pouch cells containing an NMC811 cathode (NMC811/carbon/binder with a ratio of 96.4:1.6:2.0 by weight) and a 90% graphite + 10% SiO_x_ anode (graphite + SiO_x_/carbon/binder with a ratio of 94.8:1.4:3.8 by weight) were obtained from LiFUN Technology, with a capacity of 1000 mAh. The loading is 15.4 and 7.5 mg/cm^2^ corresponding to cathode and anode, respectively. The N/P ratio is 1.2. These cells were disassembled in a nitrogen environment; then, approximately 2.5 mL of electrolyte was then injected into each cell, which were sealed afterward. To ensure proper wetting and prevent copper foil dissolution, all cells were held at 1.5 V for 12 h post injection. Subsequently, the cells were cycled in the range of 2.75–4.20 V at a C/20 rate for three cycles. The cells were then disassembled, resealed to remove gases generated during the formation process, and subjected to typical safety testing (see Section 2.5) after being charged to 4.5 V at a C/2 rate.

### 2.4. Electrochemical Testing

Long-term cycling tests were performed at ambient temperature utilizing a cell testing system (NEWARE CT-4008, Shenzhen, China), with a voltage range of 3.0–4.5 V at a C/3 rate. Rate testing was conducted within the 3.0–4.5 V range, with 5 cycles at each of the following rates: C/2, 1 C, 2 C, 4 C, and C/2. Post two initial cycles at C/2 within the 3.0–4.5 V range and charging to 4.5 V, the NMC811 cells were placed in a 60 °C chamber for high-temperature storage. The open-circuit voltage was supervised every 0.5 h over a 7-day period. Post storage period, two additional cycles were performed using the same protocol to evaluate the cell’s capacity loss during high-temperature storage.

### 2.5. Safety Testing

The electrolytes’ flammability was assessed through self-extinguishing time (SET) testing, in which a torch was applied to a container holding 0.5 mL of electrolyte. The thermal stability of the electrolyte and electrode materials was explored via Differential Scanning Calorimetry (DSC, Mettler-Toledo DSC3), with an electrode-to-electrolyte mass ratio of 1:1. The temperature range for DSC was 50–350 °C, with a heating rate of 10 °C/min. The thermal behavior of pouch cells at elevated temperature was analyzed using ARC (THT ES), where the Heat–Wait–Search mode was applied [34]. Cells were first heated to 50 °C; then, the machine started to search the cell’s self-heating at a step of 10 °C. The cells’ immunity to mechanical damage was tested through nail penetration testing, where a 3 mm diameter nail was driven into the cell at 450 mm/min, held in place for 5 min, and then retracted at the same rate. To assess the safety characteristics of pouch cells under overcharge conditions, overcharge tests were conducted at a 1C current to trigger thermal runaway.

### 2.6. Material Characterization Analysis

Atomic force microscope (AFM) testing was performed to reveal the mechanical strength of the electrode–electrolyte interface, where a machine (Bruker Dimension Icon, Billerica, MA, USA) was used and the determined positive electrodes after long-term high-voltage cycling were selected (washed by DMC to remove original electrolyte residues). Transmission electron microscopy (TEM) testing was also conducted to analyze the electrode–electrolyte interface, with a microscope (JEOL JEM 2100, Tokyo, Japan). The cathode powders were dispersed by ethyl alcohol and ultrasound for 5 min. In addition, X-ray photoelectron spectroscopy (XPS) testing using spectroscopy (Thermo Scientific K-Alpha, Waltham, MA, USA) was carried out to unveil the component of the electrode–electrolyte interface. Further, a conductivity tester (JG ST2742B, Suzhou, China) was utilized to demonstrate the conductivity of the specific electrolytes at ambient temperature.

### 2.7. Computational Calculation

Density functional theory (DFT) was based on B3LYP/6-31G* horizontal base group for structural optimization, and M062X/def2TZVP calculated the energy obtained from the single point energy. The molecular dynamics (MDs) calculation software was GROMACS-v2024.1 using the OPLSS-AA force field to generate atomic topological information. The molecules in the system were randomly constructed in boxes according to the dissolution ratio. The size of the boxes was calculated by density and optimized by the NPT ensemble. The electric field was *X*-axis uniform electric field and the temperature was 300 K.

## 3. Results and Discussion

**Figure 1:**
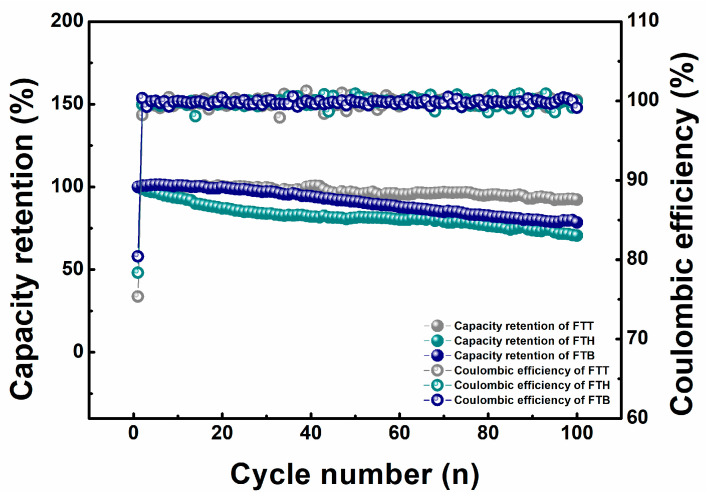
The capacity retention and Coulombic efficiency curves of the cells with the specific electrolytes cycled between 3.0 and 4.5 V at room temperature.

The long-term high-voltage cycling performance of NMC811 cells, influenced by specific electrolytes at ambient temperature, is shown in Figure 1. Figure S1 of the Supplementary File depicts the long-term cycling profiles of the pair cells added to the fixed electrolytes to show the repeatability of this work. The Coulombic efficiency and capacity retention of cells over cycling is demonstrated. As shown in Equations (1) and (2), the cell’s Coulombic efficiency (CE) is the ratio between the cell’s discharge and charge capacities to denote the cell’s reversibility, while the cell’s capacity retention (CR) is the ratio between the cell’s discharge capacities and the first-cycle discharge capacity to denote the cell’s degradation. Compared to the other two fluorinated ethers, TTE demonstrates superior compatibility with FEC and TFEC, effectively maintaining high cell performance under elevated voltage conditions. As a result, the cells achieve a capacity retention of approximately 92.4% after 100 cycles, along with an average Coulombic efficiency approaching 100%. Figure S2 shows the incremental capacity curves of the specific cells during the first and fifth cycles. In comparison to FTH cells, both FTT and FTB cells exhibit worse polarization during the first charge process. This moves the lower voltage region during the following cycles, indicating the generation of stable passivation layers. In contrast, the incremental capacity curve of FTH cells moves gradually to a higher voltage during charging, in accordance with their severe degradation at high voltages. The high-overlapping incremental capacity curves during discharging for FTT cells confirm the sound property of the passivation layer between the electrodes and electrolytes, which maintains the high performance of cells when exposed to elevated voltage, although its initial Coulombic efficiency and discharge capacity were lower than those of the others (see Figure 1 and Figure S3). The long-term cycling performance of FTT is more competitive than that of some other fluorinated electrolytes reported in the literature [35,36], as well as the commercial electrolyte [36] under similar cycling conditions. In addition, the performance of the cell added to the electrolyte in the absence of the fluorinated ether, i.e., 1.0 M FEC:TFEC (4:6 by weight), has not been significantly enhanced, in comparison to the FTT result [37]. Combining the fact that the cost of TTE is much lower than the fluorinated esters, the utilization of FTT might be more feasible.

(1) $$CE=\frac{Q_{d,i}}{Q_{c,i}}(i=1-100)$$

(2) $$CR=\frac{Q_{d,i}}{Q_{d,1}}(i=1-100)$$

**Figure 2:**
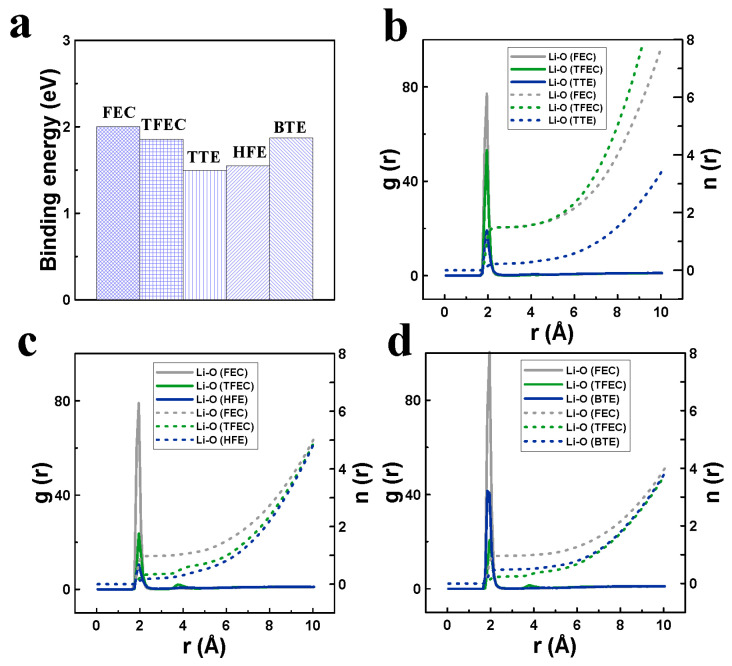
(**a**) The binding energy of the specific solvents with lithium ions; (**b**–**d**) the radial distribution function and coordination number for FTT, FTH, and FTB, respectively.

To elucidate the operating mechanism of the electrolyte containing TTE, computational calculations and material characterization analyses were conducted. As shown in Figure 2a, all the fluorinated ethers examined exhibit lower binding energy with lithium ions compared to the fluorinated esters, indicating their potential as diluents. Among them, TTE shows the lowest binding energy. Lower binding energy typically allows greater freedom of lithium ions within the electrolyte, thereby enhancing electrolyte kinetics and reducing the loss of active lithium at high voltage [38]. The high kinetics of FTT can be confirmed by the electrolyte’s ionic conductivity, as listed in Table S1 of the Supplementary File, where FTT shows a greater conductivity than the others at room temperature, although they are all lower than that of the commercial electrolyte given the high content of fluorinated components [24,39]. Figure 2b–d compare the radial distribution function and coordination number profiles of the specific electrolytes. It is evident that the lithium ion primarily coordinates with FEC in all three electrolytes, with coordination numbers of 1.49, 0.98, and 0.96 observed for FTT, FTH, and FTB, respectively. The increased coordination between FEC and lithium ions in FTT promotes its decomposition, leading to the production of an inorganic-rich electrode–electrolyte interface [5]. Previous studies have shown that a higher concentration of inorganic materials at the electrode–electrolyte interface helps stabilize the interface and suppresses undesirable reactions between the electrolyte and electrode at high voltages [40]. The improved kinetics of the FTT electrolyte, combined with a more robust electrode–electrolyte interface, contribute to the enhanced high-voltage cycling performance of the cells.

**Figure 3:**
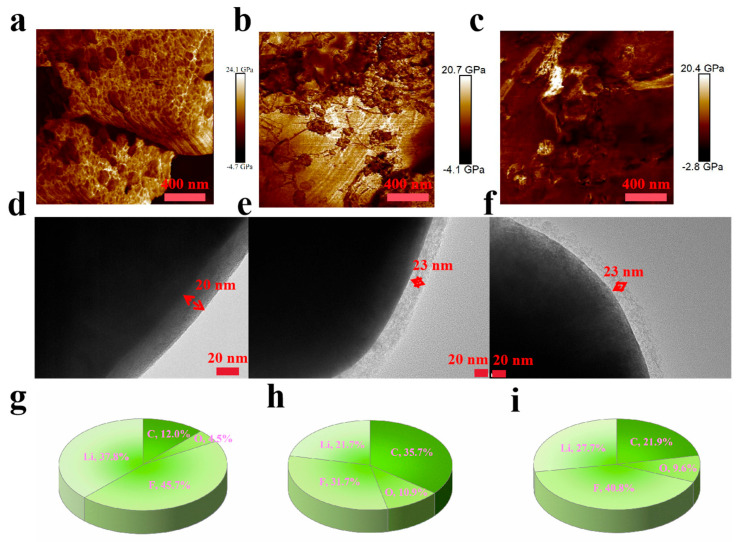
(**a**–**c**) The Young’s modulus mapping image of the cathode materials scraped from the FTT, FTH, and FTB cells after cycling, respectively; (**d**–**f**) TEM spectra of the cathode materials scraped from the FTT, FTH, and FTB cells after cycling, respectively; (**g**–**i**) element ratios of the cathode materials scraped from the FTT, FTH, and FTB cells after cycling, respectively.

The Young’s modulus mapping profiles of the cathode materials from the FTT, FTH, and FTB cells post high-voltage cycling were captured using atomic force microscopy (AFM), as shown in Figure 3a–c. The average Young’s modulus values are 8558, 8306, and 5072 MPa for FTT, FTH, and FTB, respectively. A higher average Young’s modulus indicates a strengthened electrode–electrolyte interface in the FTT cell, likely due to the inorganic-dominated interface formed by FTT, consistent with the computational calculations discussed earlier. This robust electrode–electrolyte interface contributes to the enhanced resistance of the FTT cells to high voltages. The morphology of the cathode–electrolyte interface was further analyzed by transmission electron microscopy (TEM), as shown in Figure 3d–f. Compared to FTH and FTB cells, the FTT cell exhibits somewhat thinner passivation layers, suggesting suppressed electrolyte decomposition and oxidation. The stable electrode–electrolyte interface in FTT cells effectively protects both the cathode and electrolyte from ongoing side reactions at high voltage, thereby limiting the passivation layer’s growth. Finally, the elemental composition of the cathode–electrolyte interface was uncovered by X-ray photoelectron spectroscopy (XPS), as depicted in Figure 3g–i. Notably, the FTT sample contains a significantly higher amount of inorganic elements, and the ratios of carbon and oxygen are lower compared to the FTH and FTB samples, indicating reduced electrolyte decomposition and oxidation. These XPS results align with the findings presented in Figure 2, where the increased coordination between FEC and Li^+^ in the FTT cells facilitates the production of an inorganic-rich electrode–electrolyte interface, further enhancing its stability. In conclusion, the FTT electrolyte forms a thin yet robust electrode–electrolyte interface that effectively protects both the cathode and electrolyte under high voltage, leading to outstanding high-voltage long-term cycling characteristics.

**Figure 4:**
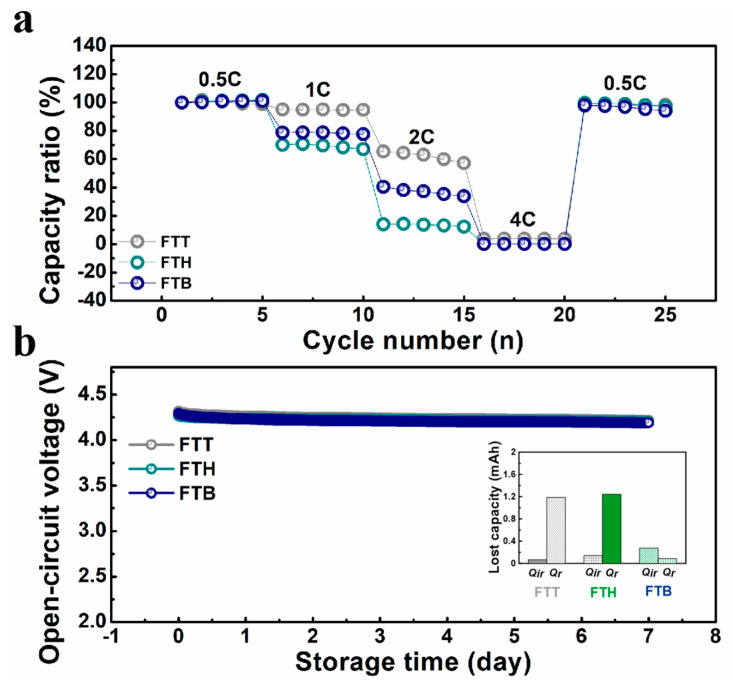
(**a**) The capacity ratio curves of the cells with the specific electrolytes cycled between 3.0 and 4.5 V at rate tests; (**b**) the open-circuit voltage curves of the according cells during high-temperature storage.

To further evaluate the electrochemical performances of the cells with varied electrolytes, rate testing was conducted and is shown in Figure 4a. The FTT cell exhibits a significantly higher capacity ratio compared to the other cells as the cycling rate increases, implying its superior high-rate potential. At the completion of the rate testing, the capacity ratio values are 98.4%, 97.2%, and 94.2% for the FTT, FTH, and FTB cells, respectively. Additionally, Figure 4b presents the variation in the cell’s open-circuit voltage during high-temperature storage. All three cells demonstrate competitive high-temperature stability, with only a gentle decline in their open-circuit voltages. The capacity loss during high-temperature storage was calculated based on the protocol outlined in the literature [35], and the results are shown in the inset of Figure 4b. The data reveal that the majority of the capacity loss for the FTT cells at elevated temperatures is reversible, and the irreversible capacity loss is lower than that of the FTH and FTB cells. Overall, the FTT electrolyte significantly enhances the electrochemical performance of the cells, offering superior characteristics compared to both FTH and FTB electrolytes.

**Figure 5:**
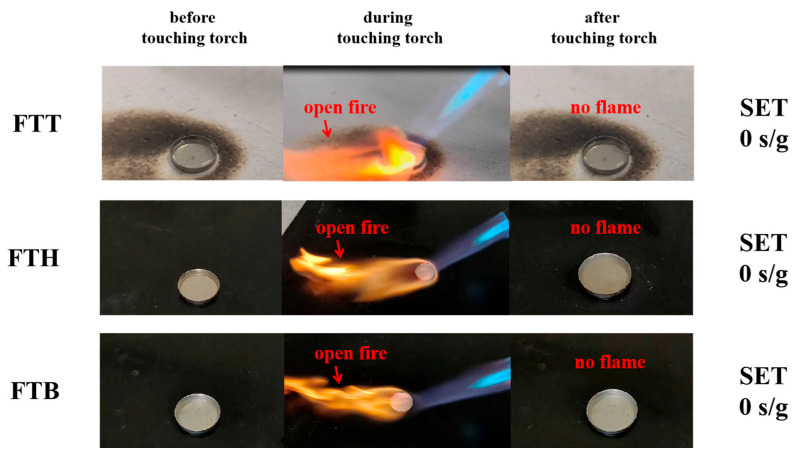
The SET tests for the specific electrolytes.

**Figure 6:**
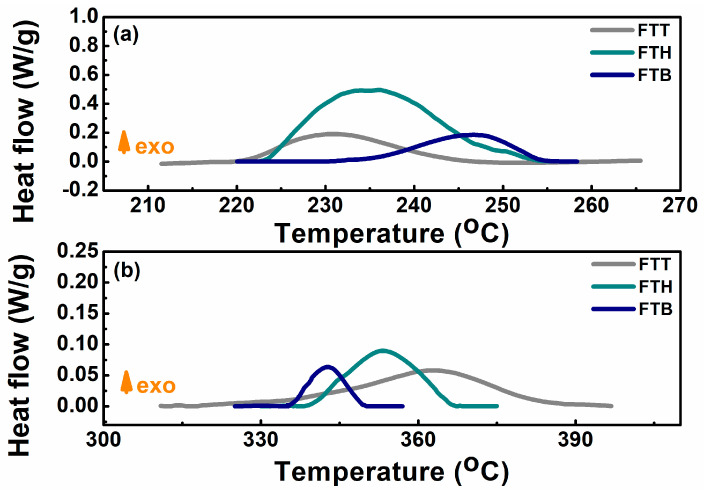
(**a**) DSC results for cathode materials in the presence of FTT, FTH, or FTB electrolytes; (**b**) DSC results for anode materials in the presence of FTT, FTH, or FTB electrolytes.

In the following, the safety properties of the specific electrolytes and the cells using them are thoroughly evaluated. The SET test results, shown in Figure 5, indicate that all three all-fluorinated electrolytes are inflammable. Upon exposure to a torch, an open flame is observed, but the flame extinguishes once the torch is removed. The presence of fluorine-free radicals in the all-fluorinated electrolytes interacts with hydrogen, oxygen, and hydroxyl radicals, disrupting the combustion process [41]. The thermal stability of the electrodes in the presence of these electrolytes was assessed utilizing DSC. As shown in Figure 6a, although the cathode materials affected by FTT begin to release heat at a relatively lower temperature, the overall heat released is less than that from the FTH- and FTB-affected systems. For the anode–electrolyte system, the FTT sample exhibits an exothermic reaction at a higher temperature than both the FTH and FTB samples.

**Figure 7:**
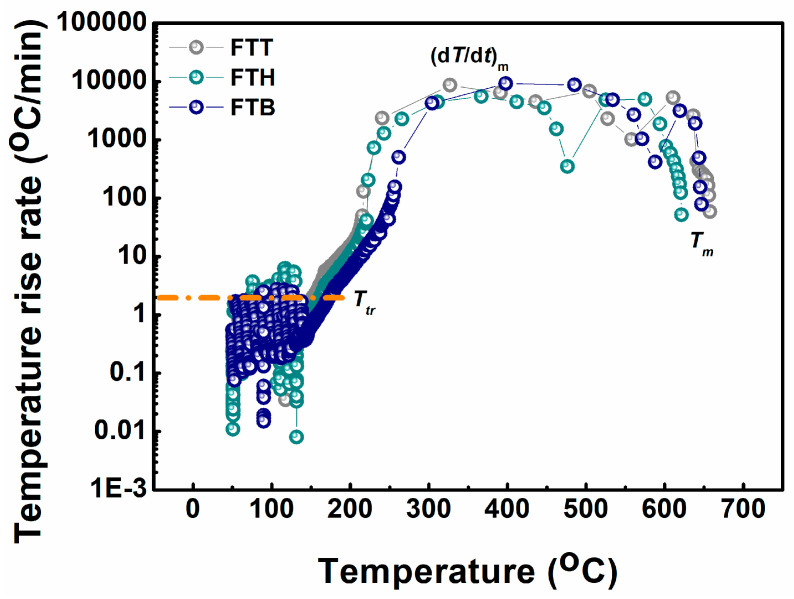
Temperature rise rate versus temperature curves for the according cells in the ARC tests.

The ARC profiles of the pouch cells with determined electrolytes are presented in Figure 7, unveiling the effect of these electrolytes on the thermal safety of cells at elevated temperatures. Some critical parameters are further extracted and displayed in Table 1. Among the samples, the FTB cell exhibits a slightly higher thermal runaway temperature (*T_tr_*) compared to the FTT and FTH cells (174.0 °C vs. 152.6 °C and 161.6 °C), but it also shows a greater peak temperature rise rate (9220 °C/min vs. 8648 °C/min and 5553 °C/min). The peak temperatures (*T_m_*) reached are 658 °C, 621 °C, and 647 °C for the FTT, FTH, and FTB cells, respectively. In summary, the FTT cells show relatively poorer safety performance under thermal abuse conditions compared to the FTH and FTB cells. This could be attributed to the earlier exothermic reaction observed in the cathode–FTT system and the greater heat release from the anode–FTT system. Even though this trade-off of FTT will not hinder its application, the difference among the three electrolytes is minor under ARC tests and there are few liquid non-aqueous electrolytes protecting cells from thermal runaway when exposed to such elevated temperatures. Appropriate additives might be considered in the future to mitigate this limitation.

**Figure 8:**
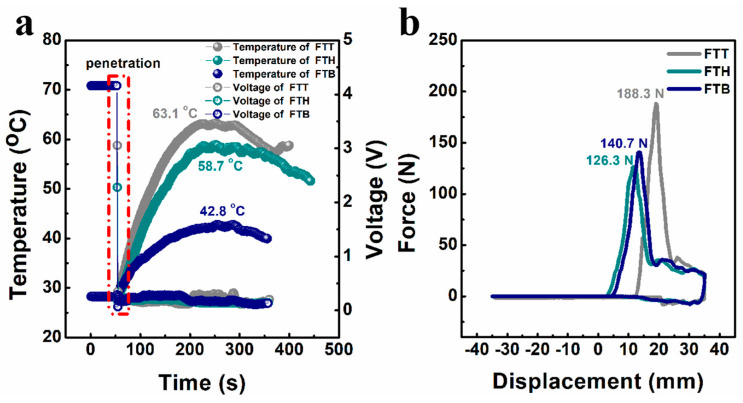
(**a**) The variation in surface temperature and open-circuit voltage of the according cells during nail tests; (**b**) nail force versus nail displacement curves of the according cells during nail tests.

The nail tests were carried out to research the resistance of the cells to mechanical abuse. As shown in Figure 8a, the open-circuit voltage of the cells drops dramatically to approximately 0 V when the nail penetrates the cells, causing an internal short circuit. Despite this, none of the three cell types experience thermal runaway during the tests. The surface temperatures of the cells peak at 63.1 °C, 58.7 °C, and 42.8 °C for the FTT, FTH, and FTB cells, respectively. This could be attributed to the superior safety characteristics of the all-fluorinated electrolytes, which effectively mitigate heat generation during internal short-circuiting. Additionally, Figure 8b illustrates the nail force as a function of nail displacement for the different cells during testing. The FTT cell requires larger force to be pierced, indicating its greater mechanical strength, which aligns with the strengthened electrode–electrolyte interface observed in Figure 3. The total work carried out by the nail was calculated, yielding values of 0.68, 0.66, and 0.67 J for the FTT, FTH, and FTB cells, respectively.

**Figure 9:**
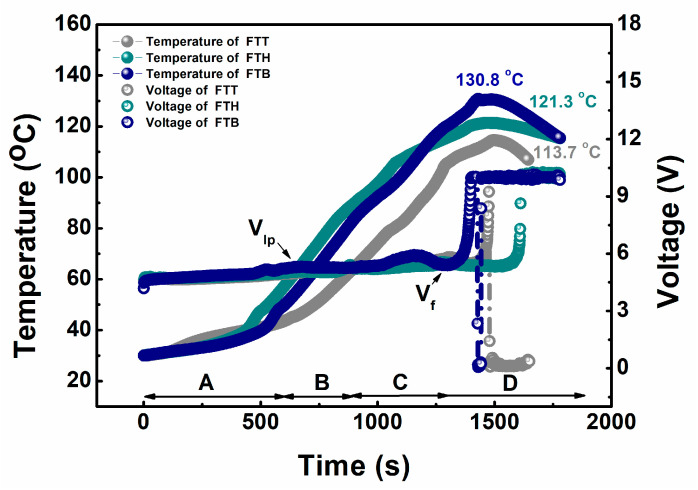
The variation in surface temperature and voltage of the according cells during the overcharge tests.

In the end, the safety features of the cells under electrical abuse scenarios were evaluated through overcharge testing. The variation in the cells’ temperature and voltage during the test is shown in Figure 9. The overcharge process can be separated into four distinct stages: at stage A, both the cell surface temperature and voltage increase steadily as the charge progresses; at stage B, the cell voltage stabilizes due to the formation of lithium plating (denoted by *V_lp_*), while the cell surface temperature continues to rise [37]; at stage C, the cell voltage initially rises again, then declines as the cell material undergoes structural collapse due to deep overcharging, while the surface temperature continues to increase; and at stage D, the cell voltage appears to rebound just before a sharp growth, indicating the approach of cell failure (denoted by *V_f_*), and then rapidly increases to the upper limit of the testing device (10 V). Subsequently, the cell surface temperature peaks at 113.7 °C, 121.3 °C, and 130.8 °C for the FTT, FTH, and FTB cells, respectively. Similar to the nail testing results, none of the cells experience thermal runaway during the overcharge tests. Notably, the FTT cell demonstrates better resistance to electrical abuse than the FTH and FTB cells, exhibiting a slower temperature rise and a lower peak temperature.

**Figure 10:**
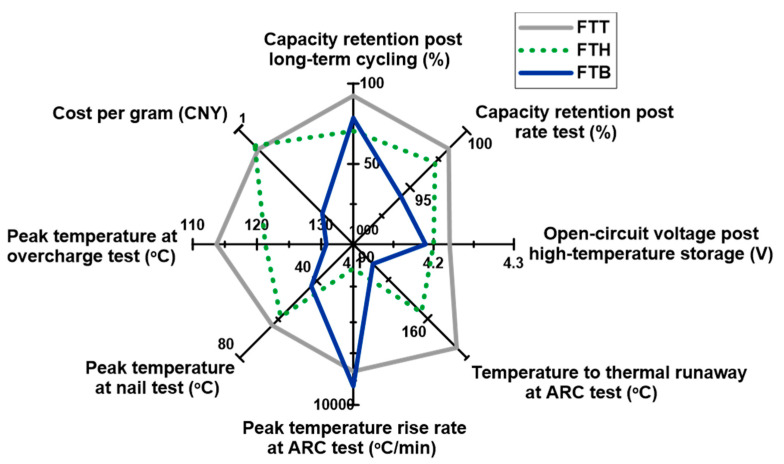
A radar plot displaying the high-voltage performances of the cells with the presence of the specific electrolytes.

Some key parameters from the electrochemical and safety tests mentioned above are extracted and presented in Figure 10 as a radar plot, providing a comprehensive evaluation of the high-voltage performances of the cells with the specific electrolytes. Capacity retention post long-term cycling, capacity retention post rate test, and open-circuit voltage post high-temperature storage are selected to represent cells’ electrochemical performances; the temperature to thermal runaway at ARC test, peak temperature rise rate at ARC test, peak temperature at nail test, and peak temperature at overcharge test are selected to represent cells’ safety performances; and the cost per gram is selected to represent the electrolyte’s price. It is evident that the electrochemical property of the FTT cell surpasses that of the FTH and FTB cells in all areas. While the safety performance of the FTT cell is not the most competitive, the differences among the three cells are relatively minor. Benefiting from the inherent safety of the all-fluorinated electrolytes, all three cell types remain immune to thermal runaway under typical abuse conditions, with the exception of elevated temperatures observed during the ARC testing. Lastly, the costs of FTT and FTH electrolytes are much lower than those of FTB, as the raw materials for FTB are more expensive and its synthesis process is more complex. Considering all of these factors, it can be concluded that TTE, when combined with FEC and TFEC, is the most optimal choice for high-performance, high-voltage cells among the selected fluorinated ethers.

## 4. Conclusions

To enable high-performance lithium-ion cells under elevated voltages, an experimental investigation was conducted to identify the optimal blend of FEC, TFEC, and common fluorinated ethers (TTE, HFE, and BTE). The electrochemical and safety properties of cells utilizing these specific ethers were systematically evaluated, and the operational mechanism of the electrolytes was analyzed, leading to the following key conclusions:

Cells utilizing TTE demonstrate superior capacity retention after long-term high-voltage cycling, owing to the enhanced kinetics of Li^+^ movement and the production of a thin, robust, inorganic-rich electrode–electrolyte interface. This interface effectively improves lithium-ion mobility, reduces active lithium loss, and inhibits side reactions of electrodes and electrolytes at high voltage. Additionally, the cells with TTE exhibit more competitive performance in both rate and high-temperature tests. As a result of the inherent safety of these electrolytes, all proposed formulations are inflammable, with an SET value of 0 s/g, and the cells containing these electrolytes show strong safety characteristics under typical abuse scenarios. Except for the ARC test, all cells avoid thermal runaway during typical abuse tests, with safety performance being similar across the different formulations. Taking the electrochemical, safety, and cost factors into account, it is concluded that the blend of FEC, TFEC, and TTE offers the most optimal solution for high-performance, high-voltage cells.

It should be noted that although fluorinated electrolytes face defects like toxicity, corrosivity, recyclability, and environmental concerns, their features enabling high safety, high conductivity, a wide electrochemical window, high energy density, etc., make them competitive for high-performance cells, especially before prior to the maturing of solid-state cell technology. Thus, fluorinated electrolytes still warrant attention. We hope this work could inspire the development of fluorinated electrolytes.

## Figures and Tables

**Table 1:** Summary of the critical parameters in ARC testing.

Cell	*T_tr_* (°C)	*dT/dt* (°C/min)	*T_m_* (°C)	Total Heat Generation (J)
FTT	152.6	8648	658	10,104
FTH	161.6	5553	621	9184
FTB	174.0	9220	647	9456

## Data Availability

The original contributions presented in this study are included in the article and Supplementary Material. Further inquiries can be directed to the corresponding author.

## Supplementary Materials

The following supporting information can be downloaded at https://www.mdpi.com/article/10.3390/ma18020274/s1. Figure S1: The repeatability of long-term cycling results for fixed cells; Figure S2: (a) The selected incremental capacity curves of FTT cells; (b) The selected incremental capacity curves of FTH cells; (c) The selected incremental capacity curves of FTB cells; Figure S3: The discharge capacity curves of the cells with the specific electrolytes cycled between 3.0 and 4.5 V at room temperature; Table S1: A list of electrolyte conductivity.
