# Supplementary material for: Optimal Blend Between Fluorinated Esters and Fluorinated Ether for High-Performance Lithium-Ion Cells at High Voltage
Source: Materials (Basel). 2025 Jan 9;18(2):274. doi: 10.3390/ma18020274 (PMC11766463; doi:10.3390/ma18020274)
Supplement: Supplementary file 1 [file materials-18-00274-s001.zip › materials-3382657-supplementary.pdf]

Supplementary Materials

Optimal Blend Between Fluorinated Esters and Fluorinated Ether for High-Performance Lithium-Ion Cells at High Voltage

Yong Sheng [1,†], Bo Liu [2,†], Junjiang He [1], Maoyong Zhi [3] and Dongxu Ouyang [2,3,*]

1 China Academy of Safety Science and Technology, Beijing 100012, China; 18910181582@163.com (Y.S.); hjj0513@mail.ustc.edu.cn (J.H.)
2 College of Safety Science and Engineering, Nanjing Tech University, Nanjing 211816, China; 202261201047@njtech.edu.cn
3 Sichuan Province All-Electric Navigation Aircraft Key Technology Engineering Research Center, Civil Aviation Flight University of China, Guanghan 618307, China; zhimaoyong@cafuc.edu.cn
* Correspondence: ouyang11@mail.ustc.edu.cn
† These authors contributed equally to this work.

Figure S1 depicts the long-term cycling profiles of the pair cells addicted with the fixed electrolytes to confirm the repeatability of the according results.

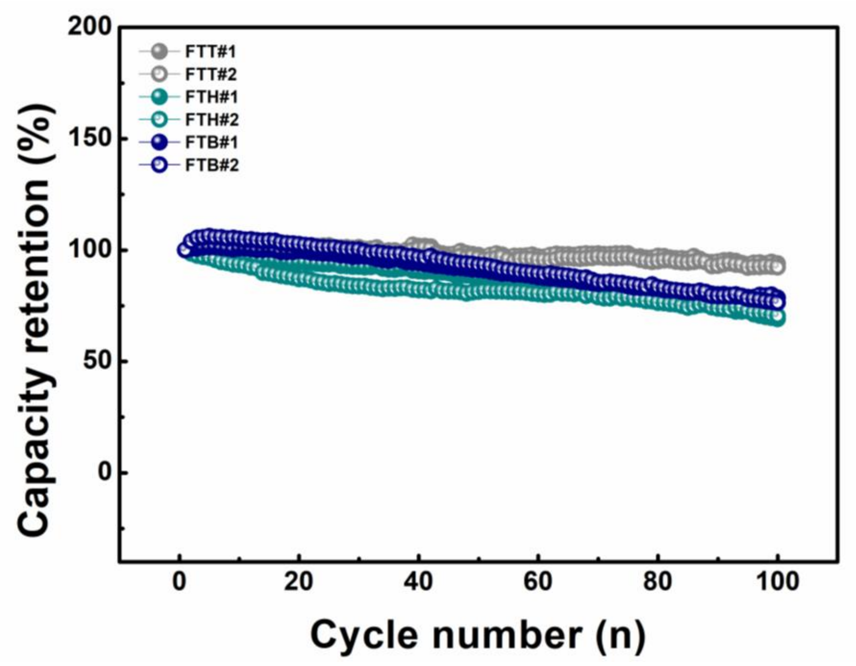

Figure S1. The repeatability of the long-term cycling results for the fixed cells.

Figure S2 shows the incremental capacity curves of the specific cells during 1st and 5th cycles.

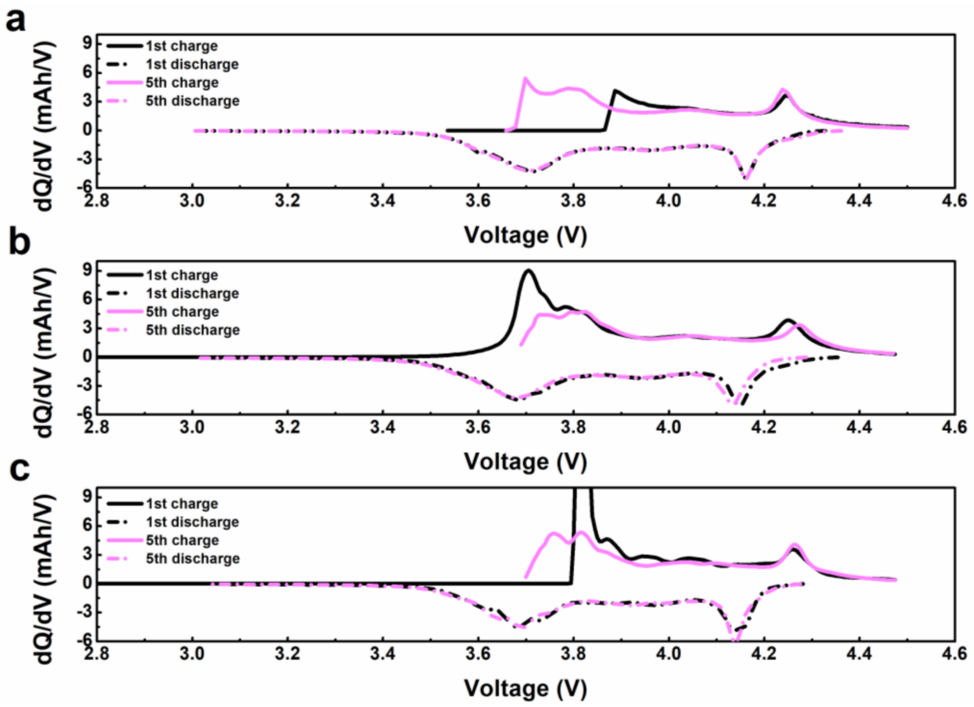

Figure S2. (**a**) The selected incremental capacity curves of FTT cells; (**b**) The selected incremental capacity curves of FTH cells; (**c**) The selected incremental capacity curves of FTB cells.

Figure S3 shows the discharge capacity curves of the cells with the specific electrolytes during the long-term high-voltage cycling.

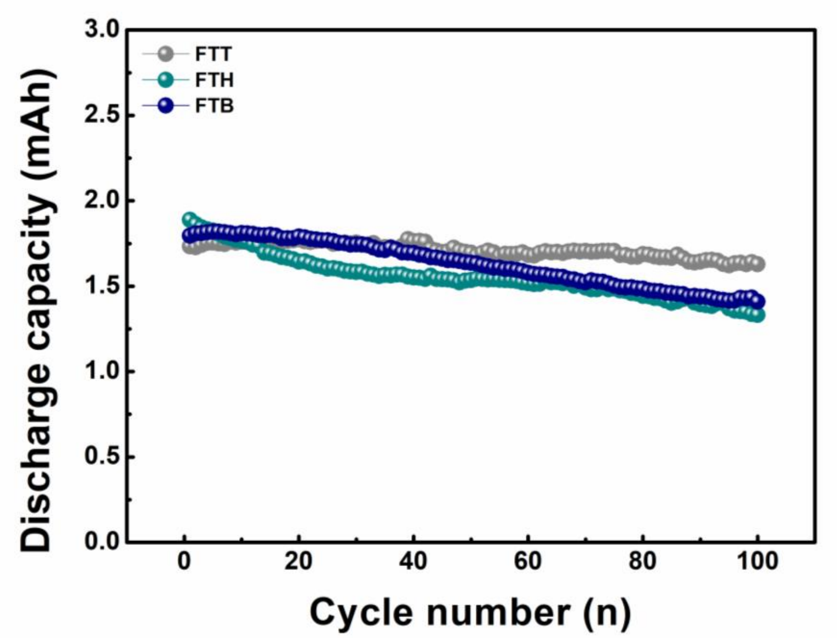

Figure S3. The discharge capacity curves of the cells with the specific electrolytes cycled between 3.0 and 4.5 V at room temperature.

Table S1 lists the conductivity of the specific electrolytes at room temperature.

Table S1. A list of electrolyte conductivity.

Electrolyte	FTT	FTH	FTB
Conductivity (mS/cm)	1.4	1.3	0.9
